# Fracture strength of non-invasively reinforced MOD cavities on endodontically treated teeth

**DOI:** 10.1007/s10266-020-00552-6

**Published:** 2020-09-04

**Authors:** René Daher, Stefano Ardu, Enrico Di Bella, Giovanni T. Rocca, Albert J. Feilzer, Ivo Krejci

**Affiliations:** 1 Department of Cariology and Endodontology, University of Geneva, 1 rue Michel-Servet, 1211 Geneva, Switzerland; 2 Department of Political Sciences, University of Genoa, Genoa, Italy; 3 Department of Dental Materials Sciences, Academic Centre for Dentistry Amsterdam (ACTA), Universiteit Van Amsterdam and Vrije Universiteit, Gustav Mahlerlaan 3004, 1081 LA Amsterdam, The Netherlands

**Keywords:** Inlay, Onlay, Cusp coverage, Endodontically treated teeth, Fiber-reinforced strip

## Abstract

The purpose of this in-vitro study was to evaluate the fracture resistance and failure mode of non-invasively reinforced endodontically treated mandibular molars. Sixty freshly extracted defect-free mandibular molars were divided into four experimental groups with extensive MOD cavities on endodontically treated teeth with different restoration types and one control group with intact teeth (*n*  = 12). The groups were as follows: “Normal”: direct resin composite; “Ring”: glass fiber-reinforced strip (Dentapreg) wrapped around buccal and lingual walls followed by direct resin composite; “Inlay”: indirect CAD/CAM resin composite inlay; “Onlay”: indirect CAD/CAM resin composite onlay; “Intact”: Intact teeth (Control). Tetric EvoCeram and Adhese Universal (Ivoclar Vivadent) were used for direct restorations and Tetric CAD (Ivoclar Vivadent) adhesively luted with Adhese Universal and Variolink Esthetic LC (Ivoclar Vivadent) were used for indirect restorations. All teeth were submitted to thermo-mechanical cyclic loading. All samples were then submitted to a compressive load until fracture. Fracture load was noted and teeth were analyzed to classify the failure mode as either catastrophic (C) or non-catastrophic (NC). No statistically significant difference was found between fracture strength of the five groups when all specimens were considered (*p* = 0.1461). Intact group showed the lowest percentage of catastrophic failures (41.67%). Ring group presents less catastrophic failures (75%) than Normal group (83.34%), and failures of indirect restorations—Inlay and Onlay—were almost all catastrophic (91.67% and 100%, respectively).

## Introduction

Fragility of endodontically treated teeth (ETT) is mostly due to accumulated tissue loss resulting from carious lesions, endodontic access cavities and canal preparation [1]. The first reason has been shown to be the most detrimental [2], since hard tissue removal at the coronal level is associated with a reduction in stiffness of up to 60% and an increase in deflection of the remaining cusps, which implies a weakening of the tooth [3]. Researchers tried to resolve this problem by either using adhesive restorations that can limit the deflection, or through redirecting lateral forces into vertical ones by covering the cusps with an indirect restoration. Despite the latter technique being the most recommended [4–8], restoration of posterior ETT remains an issue in everyday treatment decision making. This is mostly due to non-conclusive literature findings, which from one side indicate that cusp coverage is necessary to restore large defects on ETT while from the other side comparable good performance for non-cusp covering restorations is reported [9, 10]. From a practical point of view, the choice between a restoration with no cusp-coverage like a direct resin composite restoration and a cusp-covering alternative which is often done through an indirect approach, makes a significant difference to both the patient and dentist in terms of time, cost and complexity of treatment. Besides requiring the involvement of a dental lab technician and multiple treatment sessions or a chairside CAD/CAM system, indirect cusp-covering restorations can be considered more invasive since sound dental hard tissues are often removed in the preparation process. With multiple studies showing that removal of even small volumes of hard tissue is directly linked to reduced stiffness and reduced fracture resistance [11–13], non-invasive approaches should be favored. A recently published finite element analysis (FEA) study [14] has proposed the use of fiber-reinforced “rings” to wrap around the occlusal part of remaining buccal and lingual walls of large mesio-occluso-distal (MOD) cavities on ETT. The rationale was to limit the deflection of the cusps through a non-invasive method. This proof of principle study showed that mechanical consolidation of buccal and lingual walls reduced cusp deflection and stress values at the cervical level of the tooth. If proven beneficial, this technique could allow the restoration of large MOD cavities on ETT with a direct technique, without cusp reduction and further tissue sacrifice. Therefore, the aim of the present in-vitro study was to evaluate the effect of non-invasive reinforcement rings on the fracture behavior of endodontically treated mandibular molars with MOD cavities. Fiber-reinforced teeth were compared to normal resin composite restored teeth and to inlay and onlay indirect restorations. The tested null hypotheses were that fiber-reinforcing rings have no influence on (1) the fracture strength and (2) and the fracture mode of ETT.

## Materials and methods

### Teeth selection

Sixty freshly extracted human mandibular molars of similar dimensions were used for this study. A digital caliper (Mitutoyo series 551, Kawasaki, Japan) was used to exclude teeth that do not have the following dimensions: mesio-distal width of 11 mm (± 0.5 mm), bucco-lingual width of 9 mm (± 0.5 mm) and crown length of 7 mm (± 0.3 mm). The teeth were inspected under a stereomicroscope and inclusion criteria was absence of carious lesions, visible fracture lines in the root and a complete root formation. All samples were anonymously collected in accordance with the Swiss Human Research Act, article 2.

### Sample preparation

Teeth were stored in a sodium azide solution (0.2%) at 4 °C until the experiment onset. Each sample was bonded on a metallic holder (Baltec, Balzer, Liechtenstein)—in a vertical position—with light-curing composite; then, the root base was embedded with self-curing acrylic resin (Technovit, Heraeus Kulzer GmbH, Wehrheim, Germany) to complete the tooth stabilization.

### Endodontic procedure

Twelve teeth were left intact for positive control group (“Intact”), and one operator performed endodontic treatment on all remaining 48 test teeth, starting with a traditional endodontic access cavity. A diamond round bur was used to access the pulp chamber, followed by a tungsten carbide tapered bur (Endo-Z, Dentsply Maillefer, Ballaigues, Switzerland) to remove the entire roof of the pulp chamber without unnecessary mutilation of dentine. Canal preparation was performed with manual instruments until K-file 20 (Dentsply-Maillefer, Ballaigues, Switzerland), then rotary instruments (Pro Taper, Dentsply-Maillefer) to an apical diameter of 30. The canal preparation was accompanied by sodium hypochlorite 3% irrigation, and final obturation was made with the warm vertical compaction technique (Calamus, Dentsply Tulsa Dental Specialties, Johnson City, USA).

### Cavity preparation and restorative procedure

Standardized extensive MOD cavities were made on test teeth using coarse diamond burs (Cerinlay, Intensiv, Viganello, Switzerland), and the margins were finished with fine diamond burs. The cavities extended to 1 mm above the cemento-enamel junction (CEJ) in the occluso-cervical direction, and to the cusp tips in the bucco-lingual direction. All axial dentin was removed in that predefined region. The 48 test teeth were randomly divided into four groups (*n* = 12) according to the restoration technique: direct resin composite restoration (“Normal”), glass fiber-reinforced strip wrapped around buccal and lingual walls then direct resin composite restoration (“Ring”), CAD/CAM resin composite inlay restoration (“Inlay”), cusp reduction and CAD/CAM resin composite onlay restoration (“Onlay”).

For direct restorations, selective etching of enamel was made with 37% phosphoric acid etchant gel (Total Etch, Ivoclar Vivadent, Schaan, Liechtenstein) for 30 s. For the “Ring” group, additional etching of enamel 2 mm above the CEJ of the buccal and lingual walls was done on the 1 mm wide region underlying the fiber-reinforced strip. A single-component dental adhesive (Adhese Universal, Ivoclar-Vivadent, Schaan, Liechtenstein) was then applied to the dentin and to etched enamel and light-cured for 20 s with high power light-curing unit (LCU) of 1640 mW/cm^2^ (Valo Cordless, Ultradent Products, Salt Lake City, UT, USA). For “Ring” group, a 0.3 mm thick custom-made S2-glass fiber-reinforced resin pre-impregnated strip (Dentapreg, Brno, Czech Republic) was wrapped twice around the remaining buccal and lingual walls. The band was passed through the cavity in an “X” shape and a thin layer of nano-optimized flowable composite (Tetric EvoFlow, Ivoclar-Vivadent, Schaan, Liechtenstein) was used to cover the exposed fibers in the buccal and lingual, then light-cured for 20 s from buccal, lingual and occlusal. Cavities of both “Normal” and “Ring” groups were then filled with a universal nanohybrid composite (Tetric EvoCeram, Ivoclar-Vivadent, Schaan, Liechtenstein) following the anatomical layering technique. For the “Onlay” group, the buccal and lingual cusps were reduced by measuring 2 mm from the lowest point of the walls, using coarse diamond coated burs (Cerinlay, Intensiv, Viganello, Switzerland) and finished with fine-grained burs of the same shape under profuse water spray cooling. The cavity surfaces of all samples of groups “Inlay” and “Onlay” were sealed with a layer of the adhesive system (Adhese Universal, Ivoclar-Vivadent, Schaan, Liechtenstein) then light-cured for 20 s. The pulp chamber was with a universal nanohybrid composite (Tetric EvoCeram, Ivoclar-Vivadent, Schaan, Liechtenstein) and light cured for 20 s. Enamel surfaces were then exposed using fine-grained diamond burs.

Digital impressions were taken using an intraoral scanner (Cerec Omnicam, Sirona, Bensheim, Germany), and restorations were designed using a CAD software (Cerec SW 4.5.1). Inlays and onlays were then fabricated using recently-launched A2 shade resin composite CAD/CAM material (Tetric CAD, Ivoclar-Vivadent, Schaan, Liechtenstein).

### Luting procedure

**Fig. 1:**
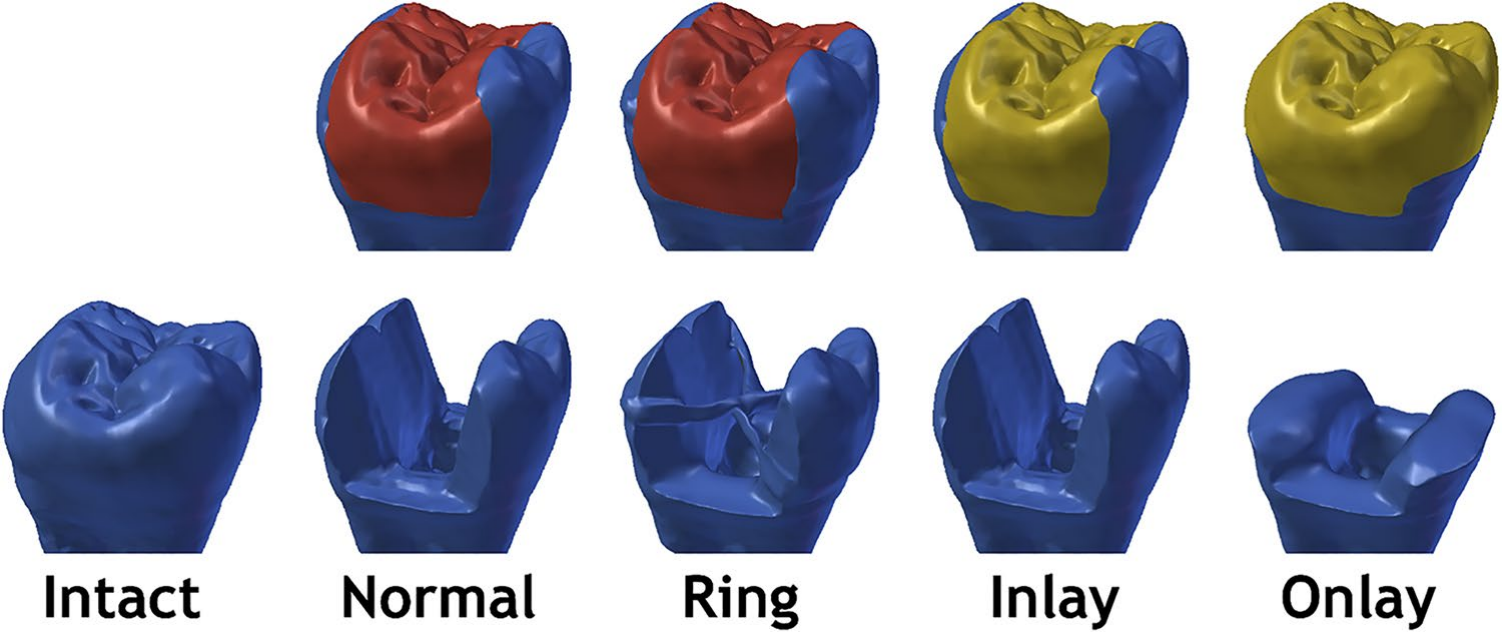
Schematic representation of the five groups of this study. Red color represents the direct resin composite restoration material and yellow color represents indirect resin composite restoration material

All restorations and adhesively sealed cavities were then sandblasted, exposed enamel was etched with 37% phosphoric acid etchant gel (Total Etch, Ivoclar Vivadent, Schaan, Liechtenstein) for 30 s and a layer of adhesive system (Adhese Universal, Ivoclar-Vivadent, Schaan, Liechtenstein) was then applied on all surfaces. A light-curing resin cement (Variolink Esthetic, Ivoclar-Vivadent, Schaan, Liechtenstein) was then applied into the cavity and the restorations were inserted followed by a removal of excess material, and light-curing for 270 s (90 s per buccal, lingual and occlusal site). Figure 1 shows the five groups of this study and Table 1 shows the materials.

**Table 1:** Materials used in this study

Material	Commercial name	Composition	Lot number
Adhesive System	Adhese Universal	2-hydroxyethyl methacrylate, Bis-GMA, ethanol, 1,10-decandiol dimethacrylate, methacrylated phosphoric acid ester, camphorquinone, 2-dimethylaminoethyl methacrylate	X20087
Etching Gel	Total Etch	Orthophosphoric acid 37%	V03984
Direct restorative resin composite	Tetric EvoCeram	Bis-GMA, TEGDMA, UDMA, barium aluminum fluoro boro silicate glass, silicon dioxide, zirconia oxide, ytterbium trifluoride	W03461
Flowable resin composite	Tetric EvoFlow	Bis-GMA, UDMA (38 wt%), barium glass filler, ytterbiumtrifluoride, highly dispersed silica, mixed oxide and prepolymers (62 wt%)	V00627
Luting agent	Variolink Esthetic LC	Bis-GMA, UDMA, TEGDMA, ytterbium trifluoride, boroaluminofluorosilicate glass, spheroidal mixed oxide, benzoylperoxide, stabilizers, pigments	X11024
Fiber reinforced band	Dentapreg	Unidirectional S2-glass fibers, dimethacrylate resins	Custom-made
CAD/CAM resin composite	Tetric CAD	Bis-GMA, Bis-EMA, TEGDMA, UDMA, barium aluminum silicate glass, silicon dioxide	X27857

*Bis-GMA* bisphenol A diglycidyl methacrylate, *UDMA* urethane dimethacrylate, *TEGDMA* triethylene glycol dimethacrylate

The margins of the restorations of all four test groups were then polished with fine diamond burs followed by polishing discs (Pop On XT, 3 M, St. Paul, MN, USA) and polishing points (Shofu inc., Kyoto, Japan).

### Thermo-mechanical testing

All samples were submitted to a chewing simulator for thermo-mechanical cyclic loading (TMCL) which consisted of 600,000 cycles of axial 49 N loads at 17 Hz delivered by a 4 mm stainless steel sphere to the occlusal surface and 3000 thermal cycles of temperature varying between 5 and 55 °C. Metallic holders were placed on a support resting on a rubber base, which induced sliding movements during mechanical cyclic loading to some extent mimic the clinical situation. Following the TMCL, a testing machine (Dyna-Mess, Prüfsysteme GmbH, Aachen, Germany) applied a continuous compressive load at 1 mm/min through a 3.7 mm diameter spherical stainless steel indenter pointed in the center of the occlusal surface until fracture.

Fracture load was noted and teeth fragments were then inspected by three different operators under stereomicroscope (SZX9, Olympus optical Co. LTD, Tokyo, Japan) to determine fracture mode. Fractures extending apical to the CEJ were considered as catastrophic while repairable fractures above the CEJ were considered as non-catastrophic.

### Statistical analysis

The different incidence of Catastrophic (C) vs non Catastrophic (NC) failures among the different groups have been checked by Fisher’s exact test. Difference among the average fracture load of the five groups has been tested using a one-way analysis of variance (ANOVA) followed by a Sheffé post-hoc test for pairwise comparisons. Normality assumptions for ANOVA were tested by means of Kolmogorov–Smirnov tests. The analyses have been run both on all the specimens and on the specimens with C failures only. Finally, a Kaplan–Meier analysis was performed on specimens with C failures only to evaluate survival functions of the five groups. All analyses have been made using STATA 15.0.

## Results

All teeth survived TMCL. Fracture mean values (SD) were 2671 N (715) for Intact, 2713 N (525) for Inlay, 2470 N (569) for Onlay, 2386 N (456) for Ring and 2211 N (359) for Normal and the numbers of catastrophic failures were 5, 11, 12, 9, 10 respectively (Table 2). Fisher’s exact test showed that the distribution of C vs NC failures is different among groups (*p* = 0.009). When the means of both catastrophic and non-catastrophic failures were considered, one-way ANOVA showed no statistically significant difference at the 95% confidence level (*p* = 0.1461). When considering catastrophic failures, mean fracture loads were 3217 N (752), 2783 N (488), 2470 N (569), 2431 N (413) and 2185 N (391) for Intact, Inlay, Onlay, Ring and Normal respectively, and a statistically significant difference was observed between groups (*p* = 0.0061) (Table 2). Scheffé post-hoc test showed a significant difference between Normal and Intact (*p* = 0.017). Survival analysis and smoothed hazard estimates showed a dominance of Intact resistance over the other groups and that Normal group results to be dominated by almost all the groups.

**Table 2:** Descriptive statistics showing average fracture load and standard deviation (SD) for all specimens, for specimens with catastrophic failures only and for specimens with non-catastrophic failures only

Group	All specimens			Catastrophic failures only			Non-catastrophic failures only			% Non-catastrophic failures
	Average load (N)	SD	*n*	Average load (N)	SD	*n*	Average loading	SD	*n*	
Intact	2671^a^	714.6	12	3217^a^	751.7	5	2282	367.0	7	58.3%
Inlay	2713^a^	525.1	12	2783^a,b^	488.5	11	1943		1	8.3%
Onlay	2470^a^	569.1	12	2470^a,b^	569.1	12				0.0%
Ring	2386^a^	456.2	12	2431^a,b^	413.3	9	2249	651.5	3	25.0%
Normal	2211^a^	359.5	12	2185^b^	391.3	10	2339	68.9	2	16.7%

Percentages of non-catastrophic failures are also presented. Common superscript letters indicate no statistically significant difference between concerned groups of the same column according the Scheffé’s test

**Fig. 2:**
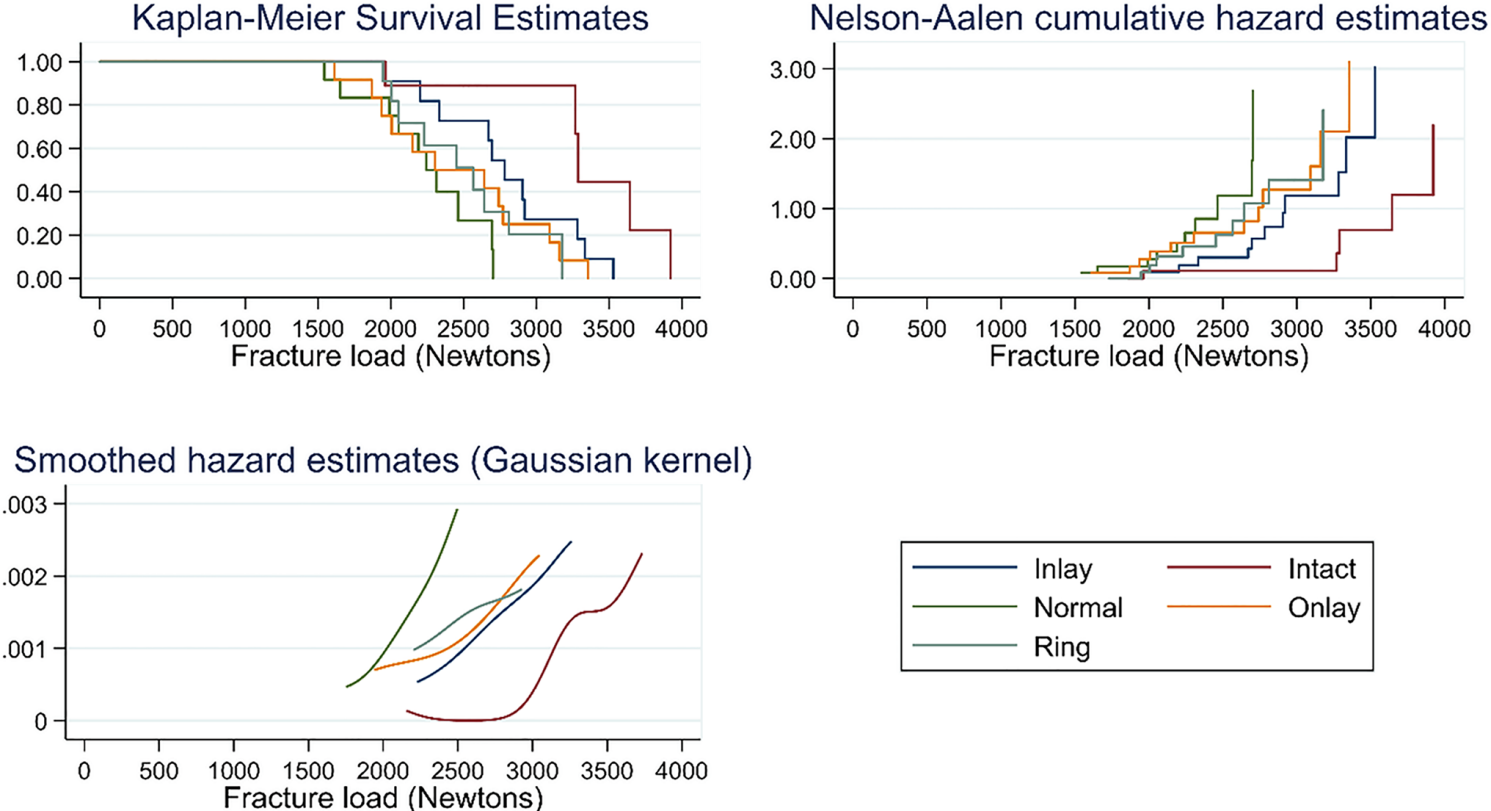
Kaplan–Meier estimates and the corresponding Nelson-Aalen cumulative and smoothed hazard estimates

Statistically significant difference among survival curves was assessed by the log-rank test (*p* = 0.002). Hazard estimates plots shows three distinct sets: Normal (the less resistant), Onlay–Ring–Inlay (almost overlapping in their resistance) and Intact (the group that reaches the highest values before catastrophic fracture) (Fig. 2).

## Discussion

This study investigated the effect of mechanically connecting buccal and lingual walls of extensive MOD cavities on ETT using fiber-reinforced strips. In terms of fracture strength, the ring-reinforced group has a higher average fracture load than Normal group, but the difference did not make statistical significance. However, when considering catastrophic failure averages, the ring-reinforced group was statistically similar to Intact group, while the Normal group was statistically different from the Intact group. Thus, the null hypothesis (1) could be partially rejected since the reinforcing technique did have a certain effect on the fracture strength of the tooth. The ring group also showed the second highest number of NC failures after intact teeth indicating that ring reinforcement does influence the fracture mode, therefore null hypothesis (2) was rejected.

**Fig. 3:**
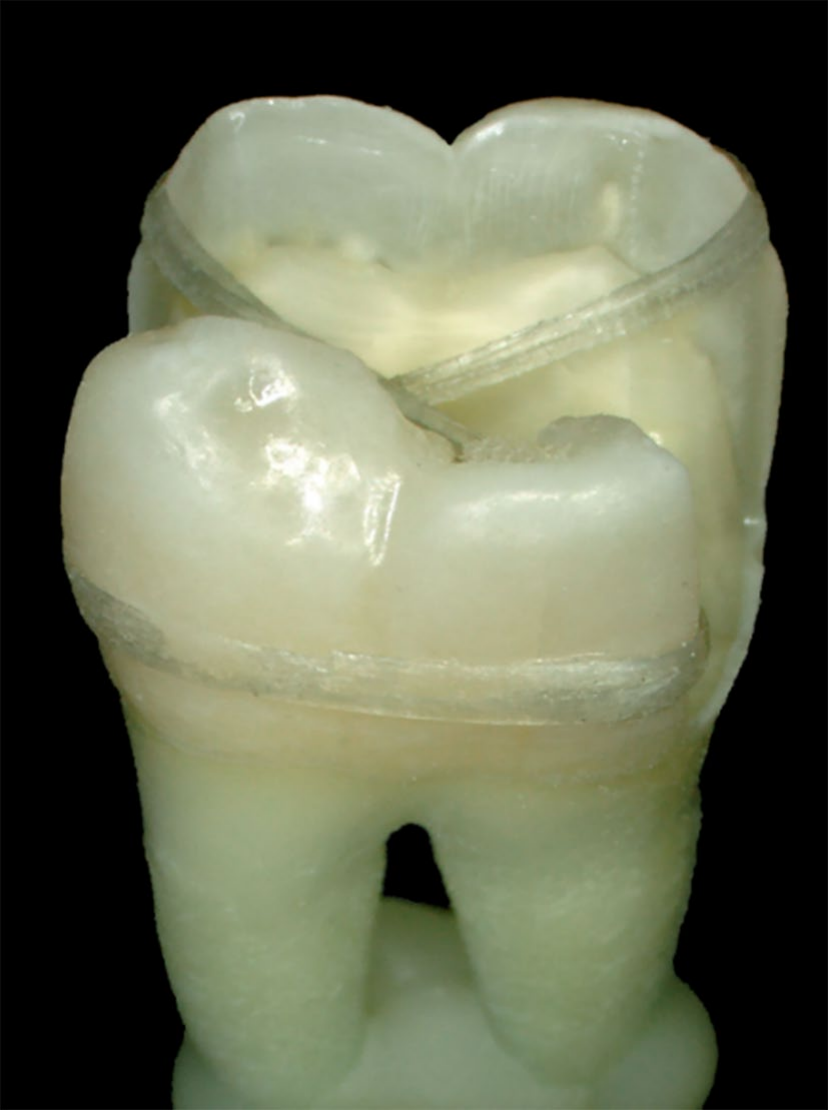
Photograph showing the fiber-reinforced ring in an “X” configuration to avoid interference with the proximal region

Mandibular molars were chosen for this test to be in agreement with the first study that first described this technique [14]. In that mentioned study, the authors propose two possible configurations of placing the fiber-reinforced strip: in a regular ring shape surrounding the crown or in a modified ring with the fibers crossing in the middle of the cavity in an “X” shape. Although this is an early in-vitro proof of concept study, multiple clinical aspects were addressed in the present research. The second configuration was used, since it is the most reasonable for clinical application to avoid interfering with adjacent teeth in the proximal areas (Fig. 3).

**Fig. 4:**
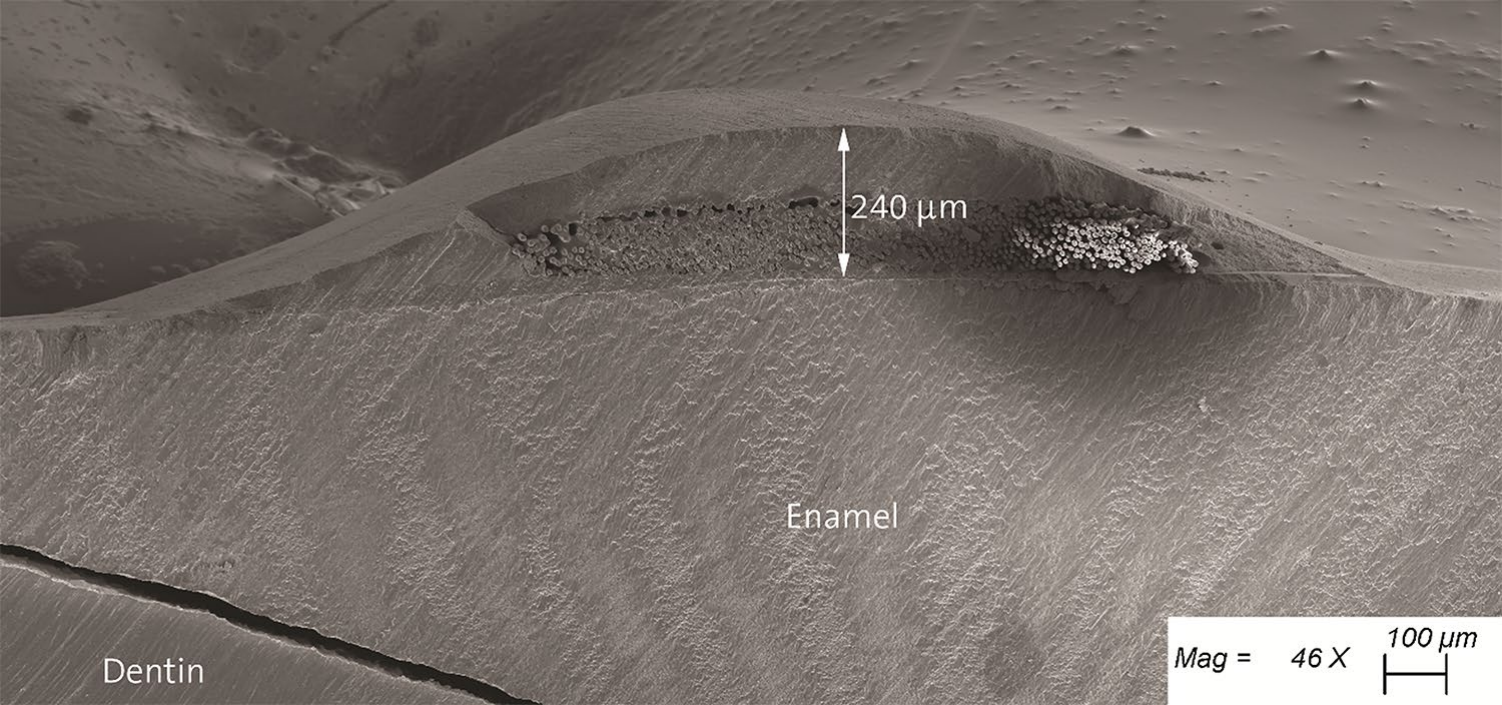
Microphotograph of a section cut showing the fiber-reinforced band in the buccal and the covering resin composite. Chips in the resin composite occurred during the sectioning and dehydration of the specimen. It is possible to observe the complete protection of the fibers, and the adaptation of the resin composite to the enamel surface

Since no closed-loop fiber-reinforced ring exists on the market, and it could be probably challenging to manufacture, the fiber-reinforced strip was wrapped twice around the tooth to first increase the number of fibers opposing to the cuspal deflection, and then to avoid detachment of the two extremities of the strip by increasing the overlapping surface of the strip. Covering the fibers in buccal and lingual with a thin layer of flowable resin composite would be clinically necessary since exposed fibers can increase plaque accumulation and gingivitis [15]. Pre-impregnated S2-glass fiber-reinforced strips were used in this study due to their high tensile strength, low plaque accumulation [16] and good fiber-adhesion to resins by silanization, which is not as efficient or even possible with alternatives like polyethylene due to their inertness and low surface energy [17–19]. One of the reasons of performing TMCL was to evaluate the aging effect of that well-established method [20–22] on that covering layer and whether fibers would be exposed post fatigue. Considering the limitations of the testing conditions compared to the clinical reality, Fig. 4 shows that the fibers remained protected after TMCL and that the final thickness of the fibers and the covering resin composite was less than 0.25 mm.

Such an addition would probably not cause discomfort for the patients. From the biological point of view, the transition between the tooth surface and the flowable resin composite was continuous, and thus no impediment of plaque control and cleaning would be caused. However, if that excess thickness turns out to be problematic, a shallow and narrow 0.3 mm groove on the buccal and lingual walls can be created to place the fibers and cover them flush with resin composite without altering the original contour of the tooth. Further investigations on the effect that this groove would have on the fracture resistance would be needed since this would become a minimally-invasive approach instead of a non-invasive one, but a certain point is that less tissues would be removed compared to current cusp reduction techniques. Cuspal deflection was shown to induce micro-cracks due to cyclic bending loads and the limited elasticity of dental hard tissues [23]. The FEA study that first evaluated the effect of non-invasively reinforcing MOD cavities with a fiber-reinforced strip [14], showed a reduction of cuspal deflection. It also showed that the stress values in the cervical area of ring-reinforced teeth were lower compared to normal restorations, by redirecting that stress into the fiber bundle. This suggested that an increased fracture strength might be expected, as seen in the results of the present study, along with a possible increase in fatigue resistance that would translate into a longer survival rate. It is important to note that the conditions applied in this test which uses a continuous compressive vertical load until fracture are different from the clinical reality [24, 25] where loads are more cyclic and where most failures occur due to fatigue. A possible reason for the limited increase in fracture strength of the Ring group in the present study, and which would not exist clinically, could be the location of the indenter that coincided right above the crossing point of the fibers. In all samples of the Ring group, the indenter came in contact with the fibers after crushing the resin composite layer covering it occlusally. This could indicate that the full reinforcing potential of the fibers was probably not reached due to the rupture caused by the indenter. Another factor for the lower values of fracture loads of the Ring group compared to the Inlay and Onlay groups could be the usage of direct resin composite which usually has a lower degree of conversion compared to homogenous CAD/CAM indirect materials, and therefore slightly lower fracture strength [26]. Despite the possible limitations, fracture load of the Ring group (2386 N) was higher than the Normal group (2211 N) and not much lower than the Onlay group (2470 N) that is considered the treatment of choice is cases of MOD cavities on ETT [27]. Fracture loads of the Inlay group (2713 N) were higher than the Onlay group (2470 N), which is in agreement with other studies that showed higher fracture resistance of inlays compared to onlays on premolars and molars [28–30]. This could be directly linked to the fact that onlay preparations are more invasive than inlay preparations. In this study, fracture load of Intact group was 2671 N (715) which is similar to results reported by other studies, like 2905 N (399) for intact lower molars by Saridag et al*.* [28] and 3048 N (905) by Dere et al*.* [31]. The slight difference can be due to the inevitable biological variability among natural teeth, and to the difference between the degree of fatigue of different teeth. In a laboratory setting and even clinically, it is difficult or almost impossible to determine the extent of the loads that the tooth was submitted to before the restorative procedure. This can also be seen in the inevitable variability that is present within each group, despite all efforts of standardization.

Mode of failure could be an even more important indicator that should be considered while examining a restorative technique, especially when no large difference is present between groups in terms of fracture strength. Ring group presented a lower percentage of catastrophic failures (75%) compared to Normal group (83.34%). Almost all failures in Inlay and Onlay groups were catastrophic (91.67% and 100%, respectively). None of the restorative techniques was able to restore the predominantly non-catastrophic failure pattern of intact teeth that showed 41.67% of non-repairable fractures. The smoothed hazard estimates show three clear regions in the graph. From a descriptive point a view, Onlay–Inlay–Ring present similar curves, with the Normal group on the lower boundary and Intact group on the higher side (Fig. 2). This could show that Ring group may perform as good as the two current recommended techniques for the restoration of MOD cavities on ETT, with the advantages of being less invasive and allowing the usage of direct techniques. Additional research is required to further investigate the potential and limitations of this technique.

## Conclusions

Within the limitations of this in-vitro study, it is possible to conclude that fiber-reinforcing rings present comparable fracture resistance to Inlays and Onlays, and that they increase the percentage of repairable fractures.
